# Clinical aspects of intraoperative radiotherapy in early breast cancer: short-term complications after IORT in women treated with low energy x-rays

**DOI:** 10.1186/1748-717X-8-95

**Published:** 2013-04-22

**Authors:** Benjamin Tuschy, Sebastian Berlit, Simone Romero, Elena Sperk, Frederik Wenz, Sven Kehl, Marc Sütterlin

**Affiliations:** 1 Department of Gynaecology and Obstetrics, University Medical Centre Mannheim, University of Heidelberg, Theodor-Kutzer-Ufer 1-3, 68167, Mannheim, Germany; 2 Department of Radiation Oncology, University Medical Centre Mannheim, University of Heidelberg, Theodor-Kutzer-Ufer 1-3, Mannheim, 68167, Germany

**Keywords:** Intraoperative radiotherapy, Intrabeam, Breast cancer, Short term complications, Morbidity

## Abstract

**Background:**

To assess postoperative complications, clinical outcome and histological findings in patients undergoing intraoperative radiotherapy with low energy x-rays for early breast cancer.

**Methods:**

We retrospectively analysed data of 208 women who underwent intraoperative irradiation during breast conserving surgery (BCS) between 2002 and 2007. Demographic, clinical and surgical parameters as well as short-term complications within the first postoperative week and histological findings were evaluated. Toxicities were assessed using the CTC/EORTC Score.

**Results:**

Postoperative complications were rare and the immediate toxicity low, without any grade 3/4 acute toxicity. The most frequent postoperative side effects were suggillation (24%) and palpable seroma (17.3%). In 78.6% of the axillary seroma and in 25% of the breast seroma a needle aspiration was inevitable. Erythema grade I-II of the breast was found in 27 women (13%); whereas in 7 patients (3.4%), mastitis was confirmed. In 57.7% of the cases, the pathological assessment revealed ductal invasive breast cancer and tumour size ranged between 0.1 and 4.5 cm (mean = 1.6 cm).

**Conclusion:**

IORT using Intrabeam^®^ during BCS is safe, although it is associated with postoperative adverse events such as seroma. These should be mentioned and explained to women in detail during the preoperative discussion. This explicitly clinical description is useful for daily clinical practice; especially for giving a detailed analysis of the postoperative side effects during preoperative counselling.

## Background

Breast cancer is the most common malignant neoplasm in women and the third cause of death in Europe [1]. Treatment of early breast cancer consists of breast conserving surgery (BCS), typically in combination with axillary sentinel lymph node biopsy (SNB) followed by external beam whole breast radiotherapy (EBRT). Furthermore, chemotherapy, endocrine and targeted therapies play an important role in the treatment of breast cancer. The therapeutic approaches chosen depend on the patient, as well as the clinical and pathological parameters of the tumour; such as stage, presence of hormone receptors and other biological characteristics [1].

Over time, radiotherapy techniques have become more sophisticated, and by applying an additional tumour bed boost of 10–20 Gy the risk of a local relapse could be further reduced [2]. In case of an externally delivered boost the risk of missing the target volume is considered to be 20-90% [2]. Intraoperative radiotherapy (IORT) is able to minimise this risk of missing the target and to shorten the interval between tumour excision and the beginning of the adjuvant radiotherapy [3].

There are various techniques for applying an IORT. Linear accelerators, brachytherapy or novel mobile devices generating fast electrons or low-energy x-rays (50 kV) are used for intraoperative irradiation [2].

At the University Medical Centre Mannheim, the mobile device Intrabeam^®^ (Carl Zeiss Surgical, Oberkochen, Germany) a miniature x-ray source with 50 kV maximum has been used for tumour bed boost radiation during BCS in selected breast cancer patients since February 2002.

Besides applying IORT as a boost, Vaydia et al. showed that IORT as a sole treatment in a low risk collective (women with primary unifocal ductal invasive breast cancer, aged 45 years or older) is non inferior to an EBRT regarding local recurrence [4].

A systematic review of scientific literature dealing with the efficacy and safety of IORT showed that IORT is not inferior to a conventional treatment. Also, the survival in patients treated with IORT seems to be similar to women who received conventional irradiation [1]. Furthermore, advantages like higher dose homogeneity, exclusion of non-affected structures from the radiation area and the avoidance of delay between surgery and radiotherapy outline IORT as a promising and probably steadily increasing alternative in the treatment of early breast cancer [1]. Beside these advantages, long term toxicity affecting quality of life negatively is very important for women undergoing BCS with IORT [5]. Sperk et al. showed that long term toxicity after IORT is low. Especially patients treated with IORT alone have about half the risk for developing higher grade toxicities as compared to standard whole breast radiotherapy [6].

Reviewing the literature, there are a few studies dealing with the late toxicity of IORT. However, studies looking at short-term complications are rare, especially after using the Intrabeam^®^ system [6-10].

Hence, this is the largest series dealing with immediate postoperative complications in women treated with IORT using Intrabeam^®^.

The aim of this investigation was to evaluate the perioperative toxicity, the clinical outcome and the histological findings in women undergoing IORT using low kilovoltage x-rays.

## Methods

Between February 2002 and April 2007, a total of 208 women were treated with IORT using the mobile device Intrabeam^®^ (Carl Zeiss Surgical, Oberkochen, Germany) during BCS at the University Medical Centre Mannheim, Germany. In 147 women (70.7%), IORT was applied as a boost irradiation while 61 patients (29.3%) received IORT without an additional EBRT.

This allocation was due to participation of women in the prospective international TARGIT-Trial, in which IORT as a sole treatment in a low risk collective was investigated [4].

The Intrabeam^®^ system is composed of a miniature (1.6 kg) x-ray source having a probe of 10cm length and 3.2 mm diameter, a set of spherical applicators from 1.5 to 5.0 cm in diameter, a carrier system and a control unit. Accelerated electrons aimed on a gold target produce a spherical radiation field with an isotropic dose distribution around the tip of the probe. Using this method low energy x-rays (50 kV) are generated. Due to the steep dose fall-off, this mobile device can be used in unmodified operating rooms. Prior to use, mechanical stability, dose rate and homogeneity of the emitted radiation were checked in detail. The patients received a perioperative intravenous single shot antibiotic treatment with 1.5 g cefuroxime. After informed consent was given from every woman in whom radiotherapy was planned, the surgical and radiotherapeutic procedures were performed as a standardized operating procedure. Histological findings were assessed at the Department of Pathology at the University Medical Centre Mannheim.

Demographic and surgical parameters as stated below were analysed retrospectively. Clinical outcomes as well as cosmetic results were evaluated every day during the first week after surgery. Toxicities were assessed using the CTC 4.0/EORTC Score. Other findings were documented without grading. This findings include: haematoma/suggillation, palpable seroma, mastitis, the necessity of therapeutic application of antibiotics, prolonged prophylactic antibiotic therapy for three days, breast induration, induration of the tumour bed, retraction of the scar, postoperative fever, pre- and postoperative blood count and the usage of postoperative pain relievers.

All data were collected in an Excel™ (Microsoft Corporation, Redmond, Seattle, USA) datasheet. After careful check for faulty entries and extreme values, the data were transferred into SPSS (SPSS^®^ version 17.0, SPSS Inc. Chicago, USA) for statistical analysis. Quantitative data were presented as median and range, qualitative data as frequencies. All computations were done using the SPSS statistics software.

## Results

In general there were no severe postoperative complications and the observed acute toxicity after IORT was low, without any grade 3/4 acute toxicity. A total of 208 women aged 30 to 95 years (mean = 63 years) with early breast cancer were treated with IORT between 2002 and 2007. The body mass index (BMI) of the study collective ranged between 18 and 57 (mean = 27); 22 women (10.6%) suffered from diabetes mellitus; 34 (16.3%) were smokers. There were no significant differences regarding short-term complications within the first postoperative week between women in which IORT was applied as a boost irradiation and the women who received IORT without additional EBRT.

Surgical parameters of the patients are presented in Table 1 in detail.

**Table 1:** Surgical parameters of women undergoing IORT

	**Study group**
	**(n = 208)**
Type of axillary surgery	
- SNB	109 (52.4%)
- ALNE (primary or after SNB)	115 (55.3%)
Duration of surgery (min)	
- < 60	1 (0.5%)
- 60 – 90	6 (2.9%)
- 90 – 120	35 (16.8%)
- 120 – 180	126 (60.6%)
- > 180	40 (19.2%)
Intraoperative antibiotic treatment	194 (93.3%)
Radiation treatment	
- IORT only	61 (29.3%)
- IORT Boost	147 (70.7%)
Duration of irradiation (min) mean	30 (10–60)

Data are given as n (%).

SNB = sentinel node biopsy, ALNE = axillary lymph node excision.

In 57.7% of patients, the pathological assessment revealed ductal breast cancer, and tumour size ranged between 0.1 and 4.5 cm (mean = 1.6 cm). In most cases, breast cancer occurred in the upper outer quadrant of the breast, in 49.4% the left breast was affected and in 43.8% the cancer occurred in the right side. The tumour localisation could not be assigned clearly to one single quadrant in 14 patients (6.7%). In 6 women (2.9%), metastases were detected by abdominal ultrasound, thoracic x-ray or bone scan during hospital stay. In 148 patients (71.2%), the assessment of the sentinel node revealed a tumour-free node. Histological findings and tumour localisations are depicted in Table 2.

**Table 2:** Tumour characteristics

**Parameter**	**Study group**
	**(n = 208)**
Primary tumour location – n (%)	
- left superior lateral	61 (29.3%)
- left superior medial	25 (12.0%)
- left inferior lateral	9 (4.3%)
- left inferior medial	8 (3.8%)
- right superior lateral	63 (30.3%)
- right superior medial	12 (5.8%)
- right inferior lateral	7 (3.4%)
- right inferior medial	9 (4.3%)
- unknown	14 (6.8%)
Tumor size (cm)	1.6 (0.1 – 4.5)
Nodal involvement	
- pN0	148 (71.2%)
- pN1mic	35 (16.8%)
- pN1a	6 (2.9%)
- pN2a	1 (0.5%)
- pN3a	1 (0.5%)
- unknown	17 (8.2%)
Metastasis	
- M0	193 (92.8%)
- M1	6 (2.9%)
- unknown	9 (4.3%)
Histology – n (%)	
- Ductal-invasive	120 (57.7%)
- Lobular-invasive	41 (19.7%)
- Tubulo/lobular-invasive	16 (7.7%)
- Other	31 (14.9%)
Grading - n (%)	
- 1	33 (15.9%)
- 2	132 (63.5%)
- 3	36 (17.3%)
- unknown	7 (3.3%)
Lymphangioinvasion	
- L0	22 (10.6%)
- L1	132 (63.5%)
- unknown	54 (26.0%)
Hormone receptors (ER; PR)	
- positive	182 (87.5%)
- negative	26 (12.5%)
HER-2/neu	
- positive	21 (10.1%)
- negative	181 (87.0%)
- unknown	6 (2.9%)
EIC	
- no	197 (94.7%)
- yes	11 (5.3%)

Data are given as n (%), mean and range.

ER = Estrogen receptor; PR = Progesterone receptor; EIC = extensive intraductal component.

In 206 women (99%), a wound drainage was inserted during surgery, and in 151 cases (72.6%) it was left for three to five days. The most frequent postoperative side effects were haematomas/suggillation and palpable seroma. Haematomas/suggillations were found in 50 cases (24%) in the breast and in 22 patients (10.6%) in the axilla. A surgical revision due to a haematoma or insufficient postoperative haemostasis was necessary in 3 cases (1.4%). 28 women (13.5%) developed a seroma located in the axilla; in 8 cases (3.8%) palpable seroma emerged in the breast. A needle aspiration was necessary in 2 cases (25%) with breast seroma and in 22 cases (78.6%) with seroma located in the axilla. Erythema grade I-II arose in 27 women (13%), none of them were classified as grade III or IV. Seventy-five patients (36.1%) received postoperative therapeutic antibiotics, in addition to the prolonged prophylactic antibiotic therapy for three days. In only 7 women (3.4%), the diagnosis of mastitis was confirmed. Fever, which was defined as elevated body temperature greater than 38.3°C measured with a tympanic thermometer, occurred in 22 women (10.6%) within the first seven postoperative days [11]. A further detailed description of postoperative events is depicted in Table 3.

**Table 3:** Outcome parameters

**Parameter**	**Study group**
	**(n = 208)**
Haemoglobin (g/dl)	
- before surgery	13.8 (9.1 – 18.0)
- after surgery (day 1)	12.7 (7.9 – 15.9)
- difference	-1.0 (-0.8 – 4.7)
Leukocytes (10^9^/l)	
- before surgery	6.8 (3.5 – 13.3)
- after surgery	8.3 (2.8 – 19.1)
- difference	+1.5 (-7.0 – 7.8)
Drainage – n (%)	206 (99.0%)
Duration of drainage (in days)	
- < 3 d	47 (22.6%)
- 3–5 d	151 (72.6%)
- > 5 d	7 (3.4%)
- unknown	3 (1.5%)
Palpable Seroma breast – n (%)	8 (3.8%)
- needle aspiration	2 (25.0%)
- no needle aspiration	6 (75%)
Palpable Seroma axilla – n (%)	28 (13.5%)
- needle aspiration once	11 (39.3%)
- needle aspiration twice	6 (21.4%)
- needle aspiration three times	5 (17.9%)
- no needle aspiration	6 (21.4%)
Haematoma/suggillation breast – n (%)	50 (24.0%)
- surgical revision	2 (1.0%)
Haematom axilla – n (%)	22 (10.6%)
- surgical revision	1 (0.5%)
Inconspicuous woundhealing– n (%)	86 (41.3%)
**Erythema Grade I-II** – n (%)	27 (13.0%)
Mastitis – n (%)	7 (3.4%)
Fever – n (%)	22 (10.6%)
Therapeutic antibiotic treatment – n (%)	75 (36.1%)
Induration toumor bed – n (%)	17 (8.2%)
Retraction of the scar– n (%)	2 (1.0%)
Postoperative pain reliever -n (%)	
- on demand	28 (13.5%)
- regular	79 (38.0%)
Nausea postop– n (%)	3 (1.4%)
Duration of hospital stay (in days)	
- < 7	12 (5.8%)
- 7 – 10	126 (60.5%)
- > 10	70 (33.7%)

Data are given as median (range) or n (%).

## Discussion

In recent years, breast cancer mortality rates in the western world have declined as a result of extensive research leading to advances in diagnostics and treatment. BCS radiotherapy, as an inherent part of interdisciplinary treatment, has become more sophisticated by delivering higher effective radiation doses without increasing side effects [1].

An additive irradiation of the tumour bed, as the area in which more than 90% of the in breast recurrences occur, reduces the local recurrence rate especially in young high risk patients [12]. This boost can by applied as an IORT, which is not inferior to the conventional treatment with respect to local control, safety and cosmetic results [1,6].

Besides the Intrabeam^®^ System, mobile linear accelerators delivering electrons at different energies (Novac7 (Hitesys Srl, Latina, Italy), Liac (Info & Tech, Roma, Italy) are being used for intraoperative electron beam radiation therapy (IOERT) in women suffering from breast cancer [10].

For these devices, collimators ranging form 3 to 12 cm were used to deliver electron beams. A radiation protection composed of a lead shielding has to be placed in the operating theatre before the beginning of radiation treatment [10].

Several investigations focused on the late toxicity and oncological aspects of IORT and IOERT in women suffering from breast cancer; they found that acute toxicity and postoperative complications are rare, especially when using the Intrabeam^®^ System [4,7,10,12-14].

A literature review finds that the most frequent side effects after IORT were seroma, wound healing disorders and fibrosis [1]. Veronesi et al. investigated a total of 590 women suffering unifocal breast cancer of less than 2.5 cm in diameter after an IOERT by using a mobile linear accelerator (Nocac7) as mentioned above [10]. In 574 women IOERT was applied as a sole radiation treatment while 16 patients received IOERT as an anticipated boost followed by external radiotherapy. Early complications were evaluated four weeks after surgery. 0.8% of these women showed postoperative haematomas or wound infections, whereas 2.5% had to undergo fine needle aspirations due to liponecrosis within one month after surgery.

Ivaldi et al. evaluated the acute toxicity after an IOERT boost of 12 Gy during breast conserving surgery followed by hypofractionated external whole breast radiotherapy (HEBRT) with a total dose of 37.05 Gy [15]. The acute toxicity was evaluated at the end of the external beam radiotherapy. Nine women (4.4%) developed liponecroses within 4 weeks, one woman within 6 weeks and one of them could not receive HEBRT because of a long lasting delay in wound healing after IOERT. Erythema and dry desquamation, as the most frequent early toxicity, were classified as mild by 67% of the patients. The peak of severe skin reactions occurred at the end of the external irradiation [15].

We published a series of 84 women treated with IORT as a boost using Intrabeam^®^ in a prospective study. Acute toxicity and the cosmetic result were evaluated after surgery at one week, and one, two and four to six months [8]. In this investigation, the mean tumour size was 15 mm and the most frequently used applicator size was 4.5 cm. One week after surgery, IORT erythema occurred in 3% of the patients and 2% of the women suffered delayed wound healing. In 2%, the diagnosis mastitis was confirmed. The most frequent complication after one week was haematoseroma, found in 6% of the patients. In a further investigation published by Kraus-Tiefenbacher et al. in which 57 women were treated with Intrabeam^®^, 5% suffered wound healing disorder and 3% developed a haematoma requiring needle aspiration. Erythema of the breast one day after surgery was observed in one case (1.7%) [12].

It is difficult to compare our results with the results of Veronesi and Ivaldi [10,15]. In these investigations patients were treated with IOERT and the immediate postoperative outcome was not evaluated at all.

Studies dealing with short-term complications after the usage of the Intrabeam^®^ system are scarce and their sample sizes are small. For example, Kraus- Tiefenbacher’s study only included 84 women (our’s included 208).

The incidence of mastitis was the same as mentioned in studies with a smaller collective. This relatively low incidence of wound infections in studies can probably be attributed to the routine use of prophylactic antibiotic treatment. Our results confirm a prospective trial by Joseph et al., which emphasises the necessity of prophylactic antibiotic treatment in patients undergoing IORT [16]. This investigation, which included 35 women, found that the rate of early breast infections could be diminished from 25% to 11% by the usage of prophylactic antibiotics.

However, the incidence of breast haematoma or seroma (17%) and axillary lymphocoele (29%) requiring additional therapy (e.g. aspiration) remains high. In our investigation, a needle aspiration was necessary in 25% of the seromas in the breast and 82% of the seromas located in the axilla. In a previous study we showed that the rate of clinically palpable seromas and seromas requiring needle aspiration was not increased after BCS with IORT compared to conventional EBRT [17]. Furthermore the high incidence of palpable axillary seromas in our investigation is not an immediate consequence of the IORT. Nevertheless, the potential necessity of a postoperative needle aspirations after breast surgery combined with IORT should be discussed in detail with the women prior to surgery. postoperative erythema after IORT our results differ to the investigation from Kraus-Tiefenbacher et al. [8]. In this investigation an erythema occurred in 3% in contrast to 13% in our study. The reason for the different incidences of postoperative complications does not seem to be the size of the tumour, which was 15 mm in the study of Kraus-Tiefenbacher et al., compared to 16 mm in our study.

In investigations of acute toxicity after IORT using Intrabeam^®^, the incidence of seroma and haematoma was lower than in our investigation. A reason for the higher incidences in our study may be the timing of clinical evaluation. In our trial, a daily assessment of the breast was performed after BCS and IORT. But in other studies, the first postoperative evaluation was performed after seven days when the initial acute phase reaction following surgery had declined. Furthermore, residues of haematomas and wound fluid in the breast can be observed immediately after surgery, but are (particularly) absorbed after a few days.

However, the comparison of the few existing studies dealing with acute toxicity after IORT seems to be difficult because different investigators had different endpoints, different classifications and different time points of evaluating the toxicities.

## Conclusion

These results verify the safety of IORT with low energy x-rays with regard to the acute toxicity. This investigation is the largest series dealing with perioperative toxicity after IORT with the Intrabeam^®^ system giving a detailed description of the immediate postoperative outcome. These results are useful for daily clinical practice, especially for pre- operative counselling.

## Competing interests

Carl Zeiss Meditec supports radiobiological research in the Department of Radiation Oncology. The remaining authors disclose no other potential conflicts of interest.

## Authors’ contributions

MS, FW and BT participated in the design of the study. SR and ES designed the database and required the clinical data. SK performed the statistical analysis of the data. SB helped draft the manuscript. All authors have reviewed the manuscript and approve of the final version to be published.
